# Locally-informed risk maps for yellow fever: a modelling study in Ghana

**DOI:** 10.21203/rs.3.rs-11296376/v1

**Published:** 2026-10-22

**Authors:** Seth Judson, Nicole Stephan, Mirava Yuson, Robert Ossom, Ben Aikins, Asabere Asante, Sofia Machuca, Pamela Quaye, Joseph Bonney, Joseph Oliver-Commey, Samuel Dadzie, Franklin Asiedu-Bekoe, Lee Schroeder, Ernest Kenu, David W Dowdy

## Abstract

Yellow fever (YF) outbreaks continue to occur in Africa and South America despite an effective vaccine. Knowing where and when certain areas are at greatest risk for YF could enable targeted allocation of resources for outbreak prevention and detection. While global risk maps of YF and other epidemic-prone pathogens exist, more granular models are needed for decision-makers. Therefore, we constructed a process for co-developing infectious disease risk maps with national experts and applied this process to model YF risk in Ghana. We collected local-level data on 14,534 patients tested for YF and eco-epidemiological covariates in Ghana from 2005–2024. We then created subnational risk maps for YF using ecological niche models that combined spatial epidemiological analysis and feedback from public health experts in Ghana. These risk maps can be used to inform targeted vaccination campaigns and diagnostic resource allocation. Our results demonstrate how local public health data can be combined with high-resolution earth observation data and expert feedback to create risk maps for infectious diseases such as YF that can directly inform subnational and national policy decisions.

